# BACE1 Inhibition Using 2’-OMePS Steric Blocking Antisense Oligonucleotides

**DOI:** 10.3390/genes10090705

**Published:** 2019-09-12

**Authors:** Madhuri Chakravarthy, Rakesh N Veedu

**Affiliations:** 1 Centre for Molecular Medicine and Innovative Therapeutics, Murdoch University, Perth 6150, Australia; M.Chakravarthy@murdoch.edu.au; 2 Perron Institute for Neurological and Translational Science, Perth 6150, Australia

**Keywords:** chemically-modified oligonucleotides, antisense oligonucleotides, Alzheimer’s disease

## Abstract

Amyloid beta-peptide is produced by the cleavage of amyloid precursor protein by two secretases, a β-secretase, beta-site amyloid precursor protein cleaving enzyme 1 (BACE1) and a γ-secretase. It has been hypothesised that partial inhibition of BACE1 in individuals with a high risk of developing Alzheimer’s disease may be beneficial in preventing cognitive decline. In this study, we report the development of a novel antisense oligonucleotide (AO) that could efficiently downregulate the *BACE1* transcript and partially inhibit BACE1 protein. We designed and synthesised a range of 2’-OMethyl-modified antisense oligonucleotides with a phosphorothioate backbone across various exons of the *BACE1* transcript, of which AO2, targeting exon 2, efficiently downregulated *BACE1* RNA expression by 90%. The sequence of AO2 was later synthesised with a phosphorodiamidate morpholino chemistry, which was found to be not as efficient at downregulating *BACE1* expression as the 2’-OMethyl antisense oligonucleotides with a phosphorothioate backbone variant. AO2 also reduced BACE1 protein levels by 45%. In line with our results, we firmly believe that AO2 could be used as a potential preventative therapeutic strategy for Alzheimer’s disease.

## 1. Introduction

Amyloid beta (Aβ)-42 peptide elevation in the brain is one of the pathological hallmarks of Alzheimer’s disease (AD) [1,2,3]. Aβ is produced by the cleavage of amyloid precursor protein by two secretases, a β-secretase, beta-site amyloid precursor protein cleaving enzyme 1 (BACE1) and a γ-secretase [4]. Since the amyloid precursor protein is cleaved first by BACE1, a rate-limiting step, BACE1 is a good therapeutic target [5]. BACE is an aspartyl protease and a type I transmembrane protein that is highly expressed in the brain and pancreas [5]. Elevated BACE1 expression and activity have been reported in post-mortem brains and the cerebrospinal fluids of AD patients [3,6]. BACE1 accumulation has also been observed around amyloid plaques in brains of AD mouse models and patients [7,8]. The *BACE1* gene is found on Chromosome 11 and includes nine exons. BACE1 has two aspartic protease active site motifs (DTGS and DSGT residues) in exons 2 and 6, respectively [5]. The *BACE1* pre-mRNA undergoes alternative splicing through the splice sites within exon 3 and exon 4 resulting in the production of protein isoforms that are 457 and 476 amino acids in length and expressed both in the brain and pancreas, respectively. However, the alternatively spliced variants of BACE1 have reduced β-secretase activity [5].

In recent years, antisense oligonucleotides (AOs) have shown great potential in developing therapies against various diseases. There have been six AOs that have been approved for clinical use for the treatment of various diseases including formivirsen, mipomersen, eteplirsen, nusinersen, inotersen, and volanesorsen. In particular, the approval of nusinersen was important as it demonstrated the potential of AOs for treating neurological diseases [9]. Although there have been no clinical trials on AOs targeting BACE1, six BACE1 inhibitors entered previously into clinical trials have failed due to liver toxicity in some cases and in others due to lack of improvement in cognitive decline [10]. Some early studies focused on AO development as research tools to better understand the role of BACE1 [10,11,12,13]; however, a systematic screening of steric blocking AO designs is not available. It was speculated that BACE1 inhibitors may need to be administered in the presymptomatic stages to patients at high-risk of developing AD, and may only need to partially inhibit BACE1 activity for reducing Aβ load slightly over a long period to have a beneficial effect [14,15]. As BACE1 partial inhibition may help in reducing Aβ load to rescue patients from cognitive decline, the development of BACE1 inhibitors that cause partial BACE1 inhibition is required. In this study, we systematically screened splice-modulating AOs targeting *BACE1* exons to identify an AO that results in partial inhibition of BACE1.

## 2. Materials and Methods

### 2.1. AO Design and Synthesis

The 2’-OMethyl (2’-OMe)-modified AO sequences on a phosphorothioate (2’-OMePS) backbone were designed and synthesised in-house using ABI Expedite^TM^ 8909 oligonucleotide synthesiser (Applied Biosystems, Foster City, CA, USA) using standard phosphoramidite chemistry at 1 µmol scale. The synthesised oligonucleotides were deprotected by treatment with 1 mL Ammonium Hydroxide (Sigma; Cat# 221228-500Ml, Castle Hill, NSW, Australia overnight at 55 °C. The oligonucleotides were then purified and desalted using illustra NAP-10 columns (GE Healthcare; Cat# 45-000-153, Springfield, QLD, Australia) according to the manufacturer’s protocol. AO2-PMO was purchased from Gene Tools. The high performance liquid chromatography (HPLC from Shimadzu, Sydney, NSW, Australia) analysis of the most efficient AOs are given in Table S5 (Supplementary Information).

### 2.2. Cell Culture and Transfection

HEK293 cells were obtained from Cell Bank Australia (kindly provided by Associate Prof. Bruno Meloni). Cells were grown and maintained in 10% Foetal Bovine Serum in Dulbecco’s Modified Eagle’s Medium (ThermoFisher Scientific; Cat# 11995073, Riverstone, NSW, Australia) in a humidified atmosphere 37 °C incubator with 5% CO_2_. Cells were maintained at 70–90% confluency and seeded in a plate or flask pre-treated with 50 µg/mL poly-D-lysine (Merck Millipore; Cat# P7886-50 mg, Bayswater, VIC, Australia) at densities shown in the Supplementary Information (Table S1), 24 h before transfection.

Next, the cells were transfected with 2’-OMePS AOs using Lipofectamine 3000 (ThermoFisher Scientific; Cat# L3000015, Riverstone, NSW, Australia) transfection reagent according to the manufacturer’s protocol at 400 nM and 200 nM for an initial screen. The best performing AOs were then transfected using the same protocol at the following concentrations: 600 nM, 400 nM, 200 nM, 100 nM, and 50 nM. Twenty- four hours after transfection, the cells were collected for RNA extraction or Western Blot. The AO2-PMO (Gene Tools, Philomath, Oregon, USA) was transfected into HEK293 cells at 100 µM and 250 µM concentrations by nucleofection method using SF Nucleofection kit (Lonza, Mt Waverley, VIC, Australia). For each treatment, 5 × 10^5^ cells were trypsinised, centrifuged, and resuspended in the nucleofection master mix as per the manufacturer’s protocol. The cells were then nucleofected with AO2-PMO using program CM- 130 by 4D Nuclofector system X-unit (Lonza Mt Waverley, VIC, Australia) using the SF Cell Line 4D-Nucleofector^TM^ X Kit S (Lonza; Cat# V4XC-2032, Mt Waverley, VIC, Australia) and seeded into five wells of the 24-well plate. Cells were collected at the 24 h, 48 h, three-day, and five-day time points after the first transfection for RNA extraction. 

### 2.3. RNA Extraction and RT-PCR

RNA was extracted from transfected cells using ISOLATE II RNA Mini kit (Bioline; Cat#: BIO-52073,Eveleigh, NSW, Australia) as per the manufacturer’s protocol. The *BACE1* transcripts were amplified using the primer sets (ordered from Integrated DNA Technologies, Singapore) shown in the Supplementary Information (Table S2) with SuperScript III One-Step RT-PCR kit (ThermoFisher Scientific; Cat# 12574026, Riverstone, NSW, Australia). The RT-PCR conditions for each primer set are given in the Supplementary Information (Table S4). Glyceraldehyde 3-phosphate dehydrogenase (*GAPDH*) was used as a loading control and the primer set (ordered from IDT), and RT-PCR conditions for *GAPDH* are given in the Supplementary Information (Tables S3 and S4), respectively. The products were then separated on a 2% agarose gel in Tris-acetate-EDTA buffer, stained with Red Safe (iNtRON Biotechnology; Cat# 21141, Burlington, MA, USA) and destained with water before being visualised with the Fusion Fx gel documentation system (Vilber Lourmat, Marne-la-Vallée, France). Densitometry was performed by the ImageJ Software [16]. The downregulation of products was determined by normalising the *BACE1* transcript levels to the loading control, *GAPDH*, and further normalised to the transcript levels from the untreated (UT) cells. A gene tool control was used as a scrambled (SCR) sequence.

### 2.4. Western Blot

Western Blot was performed on the best performing AO to evaluate the effect of the AO on the inhibition of the BACE1 protein in comparison to the scrambled (SCR) and untreated (UT) samples. Cells were lysed in lysis buffer (100 µL/ sample) containing 12% SDS, 100 mM Tris-HCl, pH 6.8, 10% glycerol with loading buffer containing 1.875 µL bromophenol blue, 4.688 µL dithiothreitol, and 1.5 µL protease inhibitor per 100 µL samples. Cell pellets were sonicated six times for 3 s pulses and denatured at 95 °C for 5 mins before snap-frozen on ice. Protein concentrations were determined to ensure equal loading by a protein gel with Coomassie blue staining. The proteins were separated on a 10% separating gel containing 400 mM Tris-HCL, pH 8.8, and 0.1% SDS, and 5% stacking gel containing 130 mM Tris-HCL, pH 6.8, 0.1% SDS, and 0.004% Bromophenol blue in a Tris-glycine-SDS running buffer before being transferred to a 0.2 µm nitrocellulose membrane (Biorad; Cat# 162-0112) in a Tris-glycine-methanol transfer buffer. The membranes were blocked in 5% skim milk Tris-buffered saline with 0.1% Tween for 1 h. The membrane was washed three times in Tris-buffered saline with 0.1% Tween for 20 mins each, and the membrane was incubated in primary antibodies overnight at 4 °C, 1:500 anti-BACE1 (Cell Signaling Technology, Cat# 5606, Danvers, MA, USA) and 1:1000 anti-GAPDH (ThermoFisher Scientific, Cat# PA1-988, Riverstone, NSW, Australia). After washing the membrane three times in Tris-buffered saline with 0.1% Tween for 20 mins each, the membrane was incubated in the secondary antibody (1:5000 anti-rabbit horse radish peroxidase, Thermofisher Scientific, Cat# 31460, Riverstone, NSW, Australia) for 1 h at room temperature before washing three times in Tris-buffered saline with 0.1% Tween for 20 mins each. The antibodies were detected using a Clarity Western ECL detection kit (Biorad; Cat# 1705060, Riverstone, NSW, Australia) according to the manufacturer’s protocol and visualised using chemiluminescence-based protocol on the Fusion Fx gel documentation system (Vilber Lourmat, Marne-la-Vallée, France ).

## 3. Results

**Figure 1:**
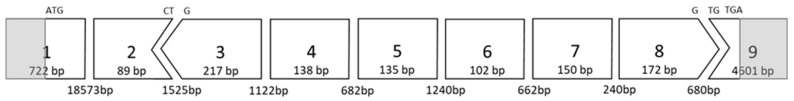
BACE1 exon map showing the size of the exons, as well as the intron size.

First, various 2’-OMePS AOs, in line with our previous work on Duchene muscular dystrophy [17,18,19,20,21,22,23], were designed and synthesised in house targeting exons (Figure 1) 2,3,4,6, and 8 of the *BACE1* gene to induce exon-skipping in the human *BACE1* transcript (Table 1). All AOs were initially screened for exon-skipping at 400 and 200 nM concentrations in HEK293 cells by incubating for 24 h using Lipofectamine 3000, Lipofectamine 2000, Lipofectin, and Lipofectamine RNAimax transfection reagents as per the manufacturer’s protocol. Twenty-four hours after transfection, the cells were collected before the total RNA was extracted, and reverse transcription-polymerase chain reaction (RT-PCR) was performed to amplify the regions of interest. The RT-PCR products were separated by gel electrophoresis on a 2% agarose gel, and the PCR products were quantified using ImageJ software. The results showed that *BACE1* transcript knockdown was achieved at various levels with all AOs targeting exon 2 and 3 (Supplementary Information Figure S1). Exon-skipping of exons 4,6, and 8 was achieved at various levels with different AOs (AO9, AO11, AO12, AO13, AO15, AO17, AO18, and AO20) targeting *BACE1* exons 4,6 and 8 (Supplementary Information Figure S2). The most efficient AOs (AO2, AO5, AO6, AO12, and AO13) targeted exons 2, 3, and 4, which were further evaluated systematically at two different series of concentration sets including 600 nM, 400 nM, 200 nM, 100 nM, and 50 nM, and 100 nM, 50 nM, 25 nM, and 12.5 nM.

### 3.1. Evaluation of the Most Efficient 2’-OMePS AOs to Induce Exon-Skipping of the BACE1 Transcript in HEK293 Cells In Vitro

**Figure 2:**
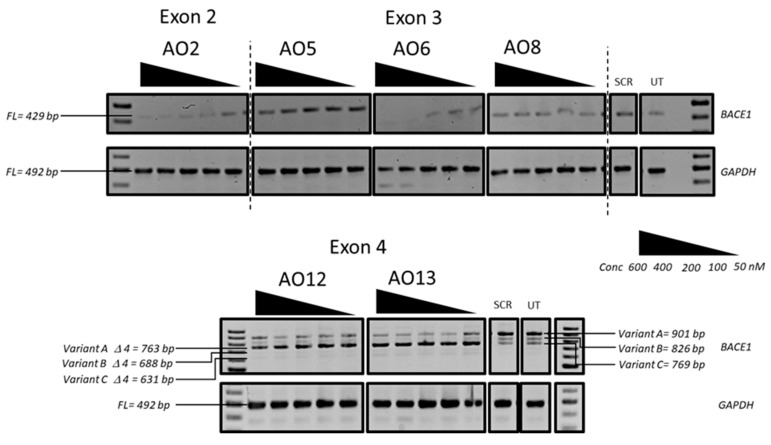
RT-PCR analysis of the *BACE1* and *GAPDH* transcripts after treatment with the best working 2’-O-MePS AOs (AO2, AO6, AO6, AO8, AO12, and AO13) targeting exons 2, 3, and 4 at 600 nM, 400 nM, 200 nM, 100 nM, and 50 nM concentrations in HEK293 cells. FL, full-length; UT, untreated; SCR, scrambled sequence; *GAPDH* was used as a loading control. The gel images were cropped to highlight the *BACE1*-specific products and the corresponding house-keeping gene control *GAPDH*. The original images are shown in Figure S3 (Supplementary Information).

**Figure 3:**
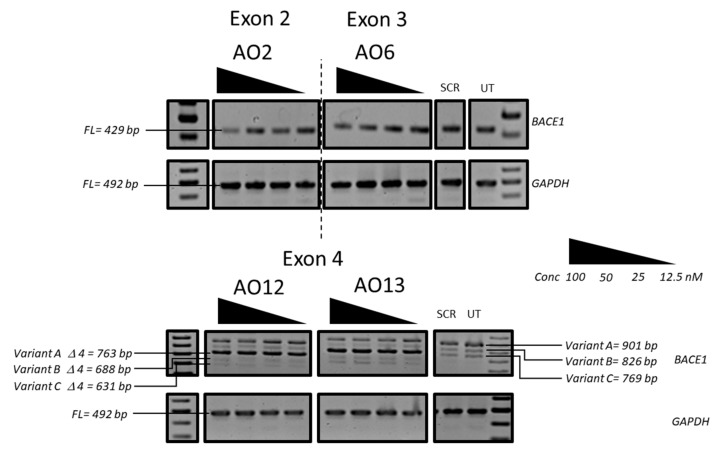
RT-PCR analysis of the *BACE1* and *GAPDH* transcripts after treatment with the best working 2’-O-MePS AOs (AO2, AO6, AO12, and AO13) targeting exons 2, 3, and 4 at 100 nM, 50 nM, 25 nM, and 12.5 nM concentrations in HEK293 cells. FL, full-length; UT, untreated; SCR, scrambled sequence; *GAPDH* was used as a loading control. The gel images were cropped to highlight the *BACE1* specific products and the corresponding house-keeping gene control *GAPDH*. The original images are shown in Figure S4 (Supplementary Information).

**Figure 4:**
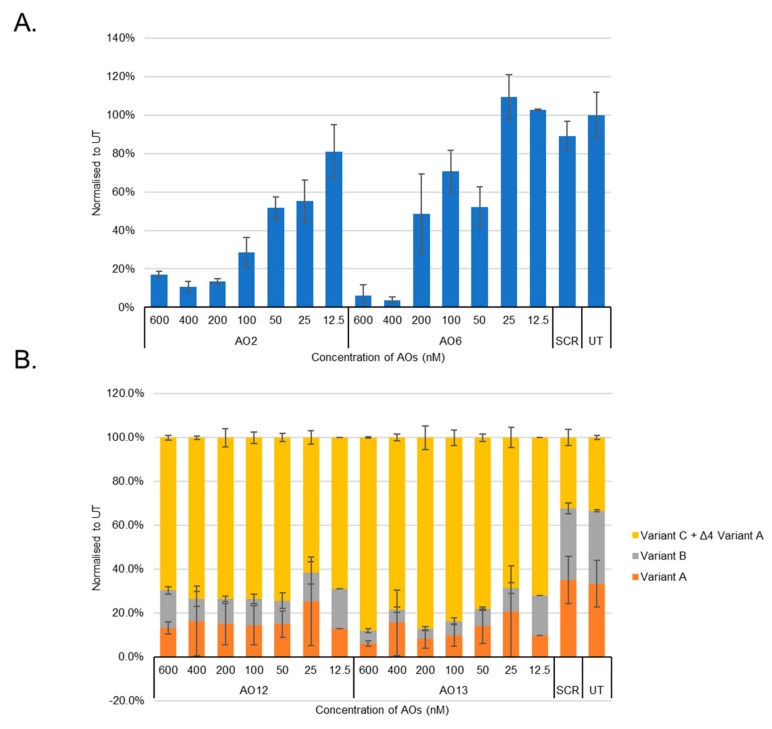
(**A**) Densitometry analysis of RT-PCR products (more than two replicates) using AO2 and AO6 showed downregulation of *BACE1* transcript in HEK293 cells in vitro. (**B**) Densitometry analysis of RT-PCR products (more than two replicates) using AO12 and AO13 showed exon-skipping of exon 4 of the *BACE1* variant A transcript in HEK293 cells in vitro. Concentrations of AOs used include 12.5 nM, 25 nM, 50 nM, 100 nM, 200 nM, 400 nM, and 600 nM. FL, full-length; UT, untreated; SCR, scrambled sequence.

We then systematically evaluated the exon-skipping efficiency of efficient AOs (AO2, AO5, AO6, AO8, AO12, and AO13; Table 1) in vitro initially at 600 nM, 400 nM, 200 nM, 100 nM, and 50 nM concentrations. The results demonstrated that AO2, AO5, AO6, and AO8 targeting exons 2 and 3 downregulated the *BACE1* transcript levels, and AO12 and AO13 that targeted Exon 4 were capable of inducing efficient exon-skipping in vitro (Figure 2). AO2 and AO6 were found to be the most efficient at downregulating the *BACE1* transcript levels in a dose-dependent manner while both AO12 and AO13 showed very high efficiency to induce exon 4 skipping of *BACE1* Variant A. Based on this, the efficacy of AO2 and AO6 and AO12 and AO13 were also further tested at lower concentrations (100 nM, 50 nM, 25 nM, and 12.5 nM). Both AO2 and AO6 showed downregulation of the *BACE1* transcript in a dose-dependent manner (Figure 3); however, densitometry analysis revealed that AO2 and AO6 were found to be the most efficient at 400 nM (Figure 4). Interestingly, although AO12 and AO13 induced exon-skipping, the dose-dependence was not as obvious (Figure 4); however, exon-skipping was observed even at 12.5 nM concentration. AO12 and AO13 were most efficient at inducing exon-skipping at 400 nM, but no concentration tested in this study could induce 100% exon-skipping. The best working AOs were determined to be AO2 and AO6, both of which showed close to 100% downregulation (Figure 4). Of the two AOs, AO2 was used for further evaluation as it showed consistent dose-dependent downregulation of *BACE1* transcript at all concentrations without affecting the loading control expression. 

### 3.2. Evaluation of the Most Efficient 2’-OMePS AOs as a PMO to Induce Exon-Skipping of BACE1 Transcript in HEK293 Cells In Vitro

**Figure 5:**
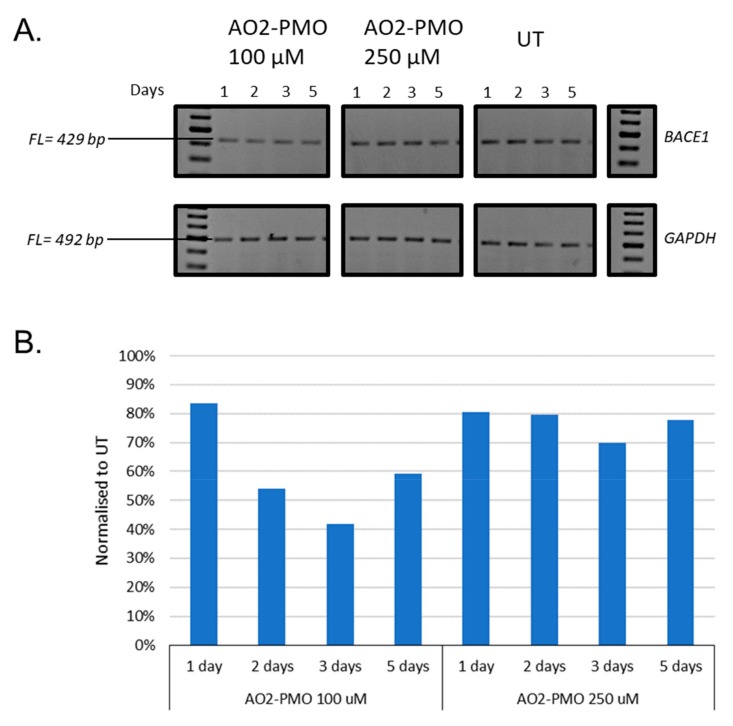
(**A**) Representative RT-PCR analysis of the *BACE1* and *GAPDH* transcripts after treatment with AO2-PMO targeting exon 2 for 1, 2, 3, and 5 days at 100 µM and 250 µM inHEK293 cells. FL, full-length; UT, untreated; SCR, scrambled sequence; *GAPDH* was used as a loading control. The gel images were cropped to highlight the *BACE1* specific products and the corresponding house-keeping gene control *GAPDH*. The original images are shown in Figure S5 (Supplementary Information). (**B**) Densitometry analysis of *BACE1* transcript downregulation in HEK293 cells in vitro.

To be clinically viable, it is important that the AOs are safe to use in humans. Towards this, PMO chemistry demonstrated excellent safety profiles in humans at very high doses. Towards this, the best working AO sequence AO2 was re-synthesised with a PMO chemistry, and called AO2-PMO, and evaluated its potential to downregulate *BACE1* transcript levels in HEK293 cells at 100 µM and 250 µM concentrations. The cells were incubated with the AO2-PMO for different time points, including 1, 2, 3, and 5 days (Figure 5). The results showed that AO2-PMO treatment downregulated *BACE1* transcript at 100 µM and 250 µM concentrations at all time points. However, BACE1 downregulation levels were much lower compared with AO2. The highest downregulation observed with AO2-PMO was around 60% after three days of incubation, whereas 89% reduction in BACE1 levels was observed after one day of incubation with the 2’-OMePS AO.

### 3.3. Evaluation of the Mechanism of Action of AO2

**Figure 6:**
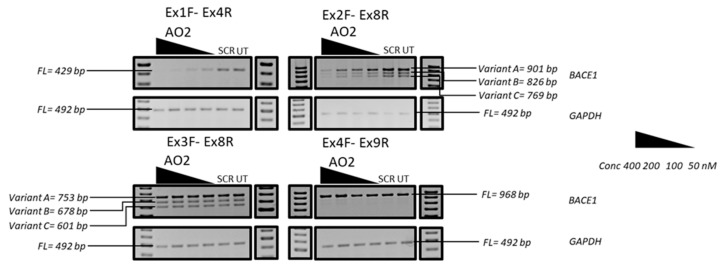
Representative RT-PCR analysis of the *BACE1* and *GAPDH* transcripts after treatment with AO2 targeting exons 2 at 400 nM, 200 nM, 100 nM, and 50 nM in HEK293 cells. Specific regions of the RNA were amplified by different primer sets. Exons 1–4 were amplified using primer set Ex1F-Ex4R, exons 2–8 were amplified using primer set Ex2F-Ex8R, exons 3–8 were amplified using primer set Ex3F-Ex8R, and exons 4–9 were amplified using primer set Ex4F-Ex9R. FL, full-length; UT, untreated; SCR, scrambled sequence; *GAPDH* was used as a loading control. The gel images were cropped to highlight the *BACE1* specific products and the corresponding house-keeping gene control *GAPDH*. The original images are shown in Figure S6 (Supplementary Information).

As AO2 was found to be the most efficient candidate that showed dose-dependent downregulation of the *BACE1* transcript, AO2 was further investigated for the mechanism of action. AO2 was designed to target the splicing enhancer region of *BACE1* exon 2 towards inducing exon 2 skipping. However, exon-skipping was not observed as predicted, and we investigated the possible degradation of the exon 2 skipped transcript by non-sense mediated decay by amplifying the other regions (exons 2–8, 3–8, and 4–9) of the AO2 treated RNA. The *BACE1* transcript was downregulated in the exon 1–exon 4 region (Figure 6) and specifically between exons 1–2 as the *BACE1* transcript was not downregulated in the exons 3–9 regions, when amplified using a variety of primers (Figure 6), which is not indicative of exon 2 skipping and subsequent degradation of the skipped product by nonsense-mediated decay.

### 3.4. Evaluation of BACE1 Protein Downregulation

**Figure 7:**
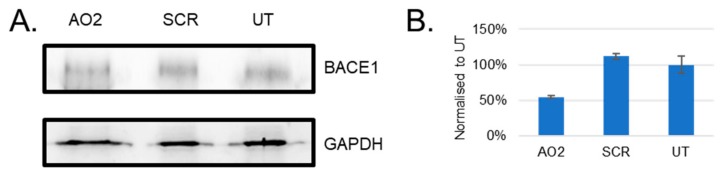
(**A**) Representative Western blot protein analysis of the BACE1 and GAPDH proteins after 24 h treatment with AO2 targeting exon 2 at 400 nM in HEK293 cells. FL, full-length; UT, untreated; SCR, scrambled sequence; GAPDH was used as a loading control. The gel images were cropped to highlight the BACE1 protein and the corresponding house-keeping protein control GAPDH. The original images are shown in Figure S7 (Supplementary Information). (**B**) Densitometry analysis of protein products showed downregulation of BACE1 protein in HEK293 cells in vitro.

The efficacy of AO2 to downregulate BACE1 protein level was evaluated in HEK293 cells after 24 h of treatment by Western blotting (Figure 7). Briefly, AO2 (400 nM) treated HEK293 cells were incubated for 24 h before collection. The cell pellet was later collected and lysed with SDS lysis buffer. The proteins were separated on a 10% separating and 5% stacking gel and transferred onto a nitrocellulose membrane. The membrane was incubated with 1:500 dilution of the BACE1 antibody overnight at 4 °C, and 1:5000 anti-rabbit HRP secondary antibody for 1 h at room temperature. The total protein loading was evaluated using a loading control, GAPDH. The membrane was incubated with 1:1000 GAPDH antibody overnight at 4 °C and 1:5000 anti-rabbit HRP secondary antibody. The antibodies were detected using a Clarity Western ECL detection kit (Biorad) using a chemiluminescence-based protocol on the Fusion Fx gel documentation system (Vilber Lourmat). The results of the Western blot analysis showed that there was 45% BACE1 protein downregulation in cells 24 h after AO2 treatment.

## 4. Discussion

A steric blocking AO that downregulates BACE1 protein expression partially has been developed in this study. AOs were designed to target regions in exons 2, 3, and 8 to induce the respective exon-skipping by blocking the binding of splicing factors which will generate a premature stop codon in the subsequent exons (in exons 3, 4, and 9). Similarly, AOs were also designed to target regions in exons 4 and 6 to induce skipping of these exons with important functional domains which are required for β-secretase activity. All of the AOs (AO1-8) targeting exons 2 and 3 of the *BACE1* transcript downregulated *BACE1* transcript at various efficiencies, of which AO2 and AO6 were found to be the most efficient; however, these AOs did not show the expected product of exon-skipping as predicted. Notably, AOs (AO9, AO11, AO12, AO13, AO15, AO17, AO18, and AO20) targeting exons 4 and 6 induced exon-skipping of the *BACE1* transcript as predicted, but AO12 and AO13 targeting exon 4 were found to be the best at inducing the exon-skipped product of 763 bp. 

This study used 2’-OMePS AOs for systematic screening, but the most efficient AO was later tested as a PMO. 2’-OMePS and PMO chemistries have been well-established for the use in vitro, as well as in vivo for developing splice modulating AOs [24,25]. Negatively charged 2’-OMePS oligonucleotides are cheap to synthesise and available commercially from several manufacturers. The PMO is a neutrally charged oligonucleotide; however, large scale production of PMO is challenging due to difficulties in synthesis which is not compatible with standard phosphoramidite chemistry. Unlike 2’OMePS chemistry, the PMO chemistry also has shown an excellent safety profile and is used clinically. Therefore, although 2’-OMePS chemistry is used for screening purposes, the most efficient AOs are later tested with a PMO chemistry. In our study, the best 2’-OMePS AO (AO2) efficiently downregulated the *BACE1* transcript but, when this sequence was tested as a PMO, it was found to be less efficient. The AO2 sequence is a G-rich oligonucleotide sequence. Therefore, we speculate that the difference observed in the efficiency of the 2’-OMePS AO and the PMO AO may be due to the PMOs forming G-quadruplex structures, while the 2’-OMePS sequence does not, which may explain the lower efficiency of the same sequence with a PMO chemistry. 

The AOs in this study were designed to target and skip exons with important functional domains by inducing premature stop codons to inhibit the expression of functional BACE1 protein. However, the most efficient AO candidate, AO2, showed downregulation of *BACE1* mRNA. We predicted that AO2-mediated *BACE1* mRNA downregulation might be due to nonsense-mediated decay, a mechanism proposed for mRNA degradation by the induction of a premature stop codon [26]. Amplification of the isolated RNA after the treatment with AO2 showed that the downregulation of *BACE1* mRNA was seen only in the exon 1–exon 2 region, but showed normal expression in the regions between exons 3–9 indicating that AO2-mediated *BACE1* transcript downregulation may not be through nonsense-mediated decay. We speculated that the potential reason for the downregulation of the exon 1–2 region might be due to a steric block imposed in this region that ultimately inhibits the translation. BACE1 protein analysis after AO2 treatment of HEK293 cells shows that there is a reduction in BACE1 level at 24 h (around 60% compared to UT). BACE1 has a half-life of over nine hours in cultured cells [5]; therefore, most of the inhibition is seen 24 h after AO2 treatment. Although we achieved close to 100% inhibition of the *BACE1* mRNA, we could not see a similar level of BACE1 protein inhibition (only 45% inhibition at protein level). This may be due to the regulation of BACE1 protein levels by mechanisms both at the transcriptional, translational, and post-translational levels [27]. As studies have shown that 5’-UTR region of the BACE1 has a role in regulating the BACE1 protein levels [27], reducing *BACE1* mRNA may only have a small role in regulating BACE1 protein level. The complete suppression of BACE1 may not be possible, but a partial reduction of BACE1 has been shown to improve amyloid neuropathology suggesting that a complete reduction of BACE1 is not required for beneficial effects [5,15,28]. Furthermore, BACE1 also has other substrates, and therefore, complete elimination of BACE1 may have deleterious effects [15,28].

## 5. Conclusions

We have screened various AOs designed to induce *BACE1* splice modulation. One potential candidate named AO2 targeting exon 2, potentially by steric blocking mechanism, was found to be the most efficient in inhibiting BACE1 expression at the RNA and protein level in HEK293 cells. Of the two chemistries evaluated, the 2’-OMePS chemistry was found to be far more efficient compared with the PMO chemistry, yielding close to 90% *BACE1* transcript downregulation and resulted in 45% downregulation of the BACE1 protein. Although further validation of AO2 in vivo and its effect on Aβ production is required to ensure the applicability of this molecule towards the clinical benefits in mitigating AD, we believe that partial inhibition of BACE1 protein levels achieved in this work could be used as a potential preventative strategy for people at high-risk of developing AD.

## Figures and Tables

**Table 1:** AO sequences targeting exons 2,3,4,6, and 8.

AO Coordinates	Target	Sequence(3′--> 5′)	AO Number
BACE1 1E2A(+ 10 + 34)	Exon 2	AGTTACTGCTGCCTGTATCCACCAG	AO1
BACE1 1E2A(+ 38 + 62)	Exon 2	AAGGGGTGGGGGGCAGCACCCACTG	AO2
BACE1 1E2A(+ 65 + 89)	Exon 2	AGCTGCCTCTGGTAGTAGCGATGCA	AO3
BACE1 1E3A(+ 16 + 40)	Exon 3	CACATACACACCCTTCCGGAGGTCC	AO4
BACE1 1E3A(+ 41 + 65)	Exon 3	CTTCCCACTTGCCCTGGGTGTAGGG	AO5
BACE1 1E3A(+ 89 + 113)	Exon 3	TGACGTTGGGGCCATGGGGGATGCT	AO6
BACE1 1E3A(+ 141 + 165)	Exon 3	TTGATGAAGAACTTGTCTGATTCAG	AO7
BACE1 1E3A(+ 193 + 217)	Exon 3	CCTGGCAATCTCAGCATAGGCCAGC	AO8
BACE1 1E4A(+ 1 + 25)	Exon 4	AGAAAGGCTCCAGGGAGTCGTCAGG	AO9
BACE1 1E4A(+ 31 + 55)	Exon 4	GAACGTGGGTCTGCTTTACCAGAGA	AO10
BACE1 1E4A(+ 61 + 85)	Exon 4	CACCACAAAGCTGCAGGGAGAAGAG	AO11
BACE1 1E4A(+ 88 + 112)	Exon 4	CTTCAGACTGGTTGAGGGGGAAGCC	AO12
BACE1 1E4A(+ 114 + 138)	Exon 4	CATGCTCCCTCCGACAGAGGCCAGC	AO13
BACE1 1E6A(+ 4 + 28)	Exon 6	TGTCCACAATGCTCTTGTCATAGTT	AO14
BACE1 1E6A(+ 36 + 60)	Exon 6	TTTCTTGGGCAAACGAAGGTTGGTG	AO15
BACE1 1E6A(+ 75 + 99)	Exon 6	GGAGGCTGCCTTGATGGATTTGACT	AO16
BACE1 1E8A(+ 6 + 30)	Exon 8	CTGCGGAAGGATGGTGATGCGGAAG	AO17
BACE1 1E8A(+ 33 + 57)	Exon 8	AAACTTGTAACAGTCGTCTTGGGAC	AO18
BACE1 1E8A(+ 63 + 87)	Exon 8	AACAGTGCCCGTGGATGACTGTGAG	AO19
BACE1 1E8A(+ 109 + 133)	Exon 8	CCCGATCAAAGACAACGTAGAAGCC	AO20
BACE1 1E8A(+ 148 + 172)	Exon 8	CATGGCAAGCGCTGACAGCAAAGCC	AO21

## Supplementary Materials

The following are available online at https://www.mdpi.com/2073-4425/10/9/705/s1, Figure S1: The RT-PCR products after treatment with AO1, AO2, AO3, AO4, AO5, AO6, AO7, and AO8 is shown here. Figure S2: The RT-PCR products after treatment with AO9, AO10, AO11, AO12, AO13, AO14, AO15, AO16, AO17, AO18, AO19, AO20, and AO21 is shown here. Figure S3: The RT-PCR products after treatment with AO2, AO6, AO12, and AO13 is shown here. Figure S4: The RT-PCR products after treatment with AO2, AO6, AO12, and AO13 is shown here. Figure S5: The RT-PCR products after treatment with AO2-PMO. Figure S6: The RT-PCR products after AO2 treatment amplified using different primer sets Figure S7: The western blot membranes after AO2 treatment incubated with anti-BACE1 antibody (top membrane) and anti-GAPDH antibody (bottom membrane). Table S1: The seeding density of HEK293 cells used for different assays., Table S2: The primer sets used to amplify *BACE1* transcript., Table S3: The primer sets used to amplify *GAPDH* transcript., Table S4: The PCR conditions for each primer set. Table S5: The HPLC analysis of the most efficient AOs (AO2, AO5, AO6, AO8, AO12, and AO13).
