# Supplementary material for: BACE1 Inhibition Using 2’-OMePS Steric Blocking Antisense Oligonucleotides
Source: Genes (Basel). 2019 Sep 12;10(9):705. doi: 10.3390/genes10090705 (PMC6770983; doi:10.3390/genes10090705)
Supplement: Supplementary file 1 [file genes-10-00705-s001.pdf]

Supplementary Information

BACE1 inhibition using 2′-OMePS steric blocking antisense oligonucleotides.

Madhuri Chakravarthy and Rakesh N Veedu

Results

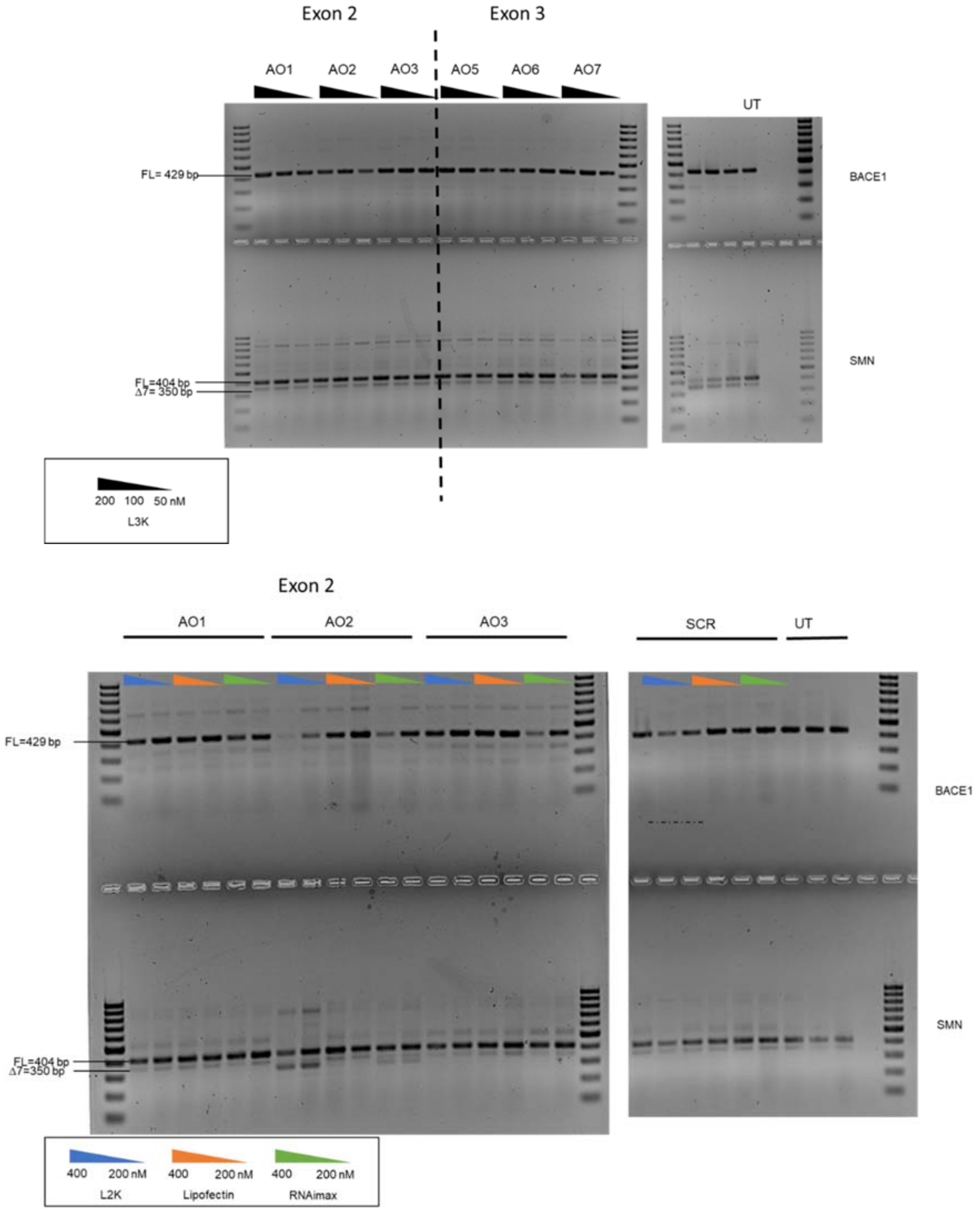

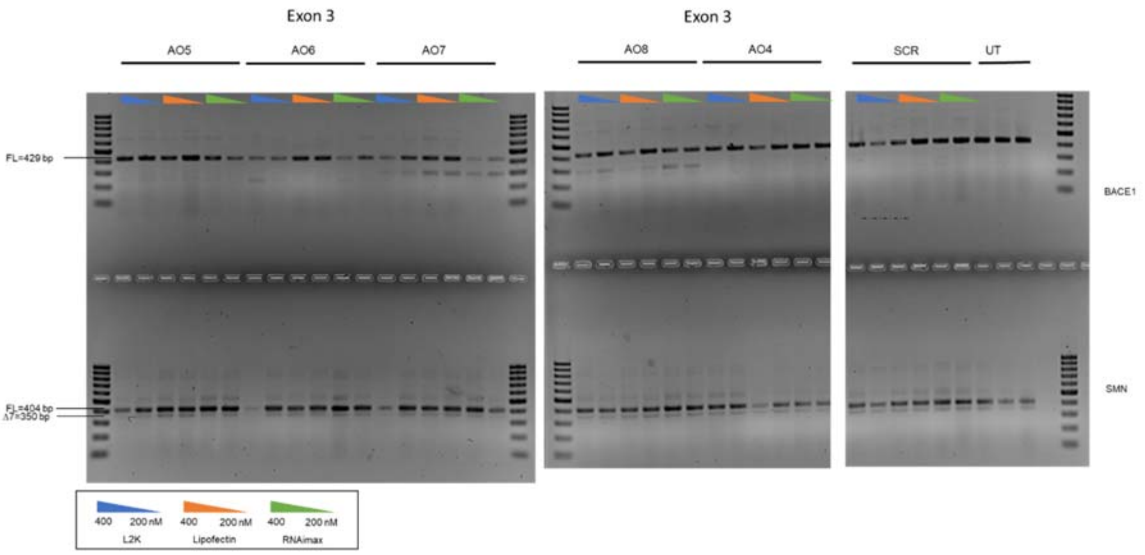

Figure S1. The RT-PCR products after treatment with AO1, AO2, AO3, AO4, AO5, AO6, AO7, and AO8. The AOs were treated using a variety of transfection reagents including Lipofectamine 3000, Lipofectamine 2000, Lipofectamine RNAimax, and Lipofectin according to the manufacturer's protocol. FL, full-length; *SMN* was used as a loading control; SCR, Scrambled or Gene tools control was used as a control. .

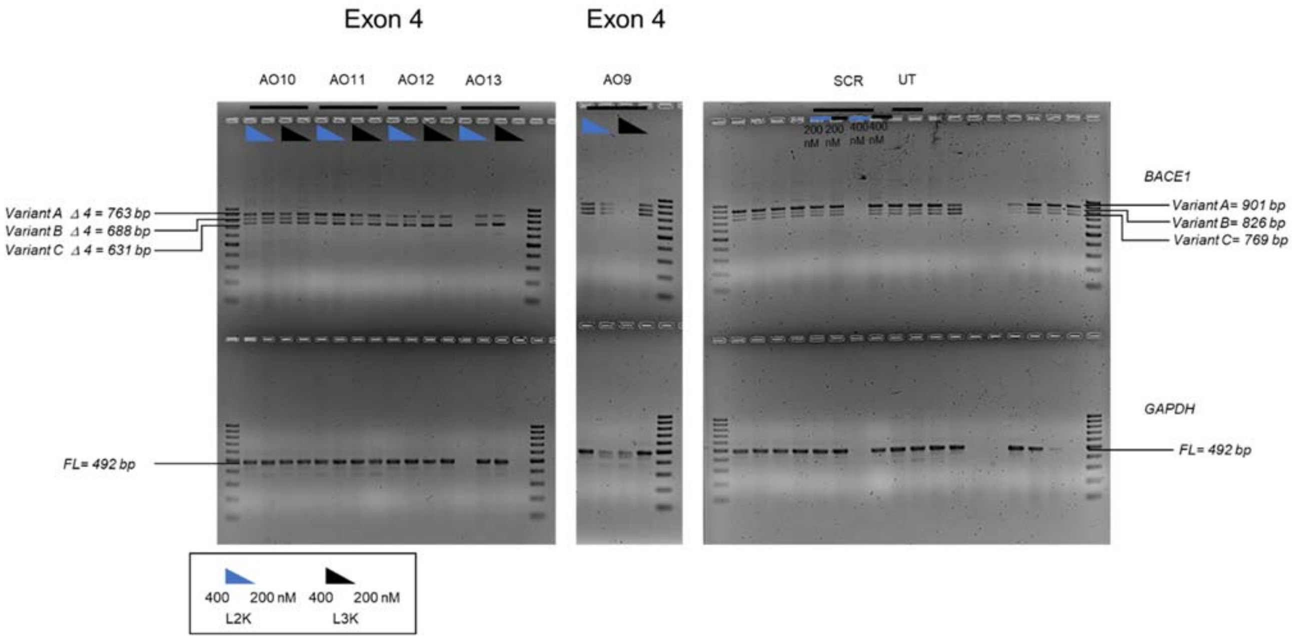

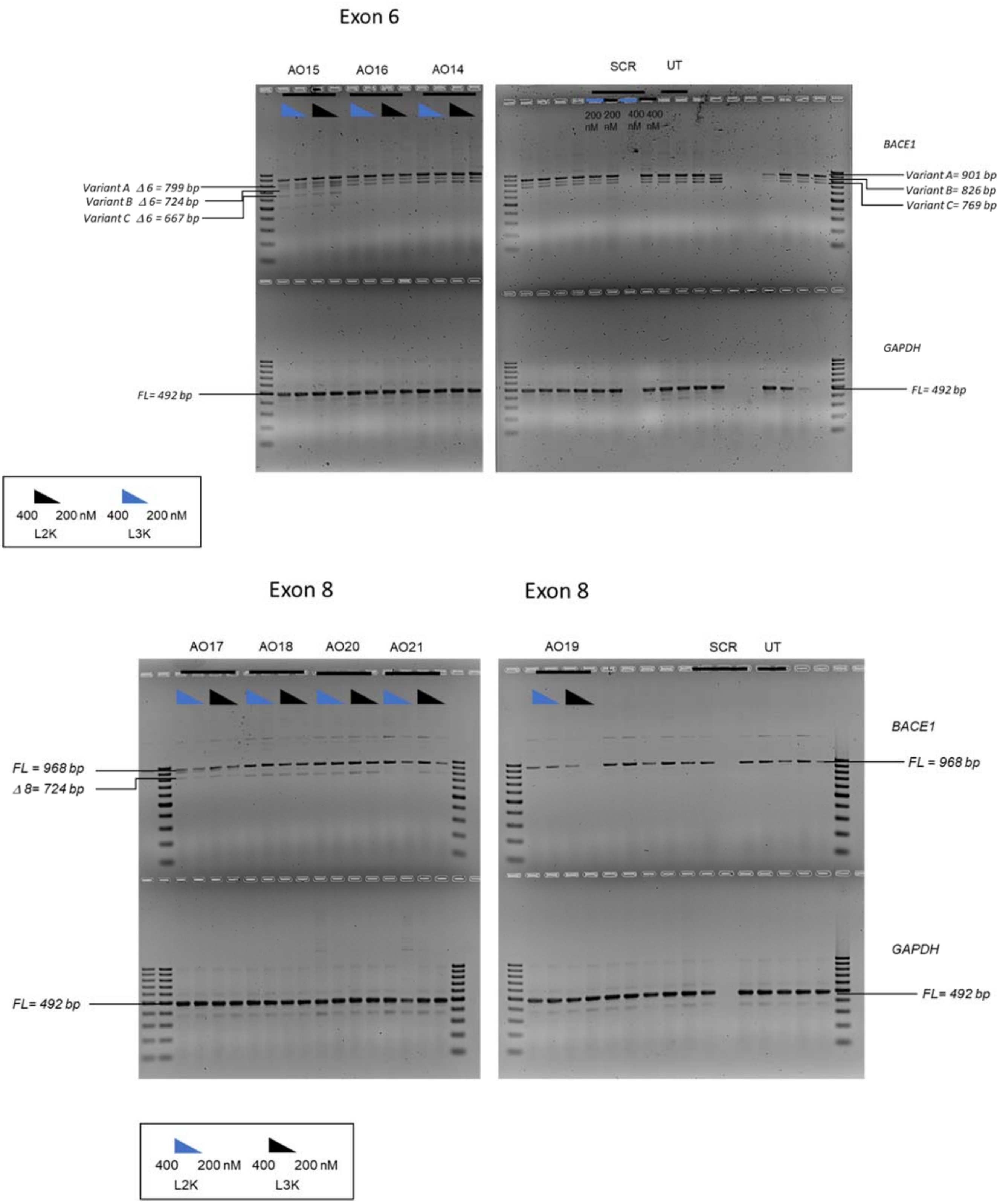

Figure S2. The RT-PCR products after treatment with AO9, AO10, AO11, AO12, AO13, AO14, AO15, AO16, AO17, AO18, AO19, AO20 and AO21. The AOs were treated using a variety of transfection reagents including Lipofectamine 3000 and Lipofectamine 2000 according to the manufacturer's protocol. FL, full-length; *GAPDH* was used as a loading control; SCR, Scrambled or Gene tools control was used as a control. .

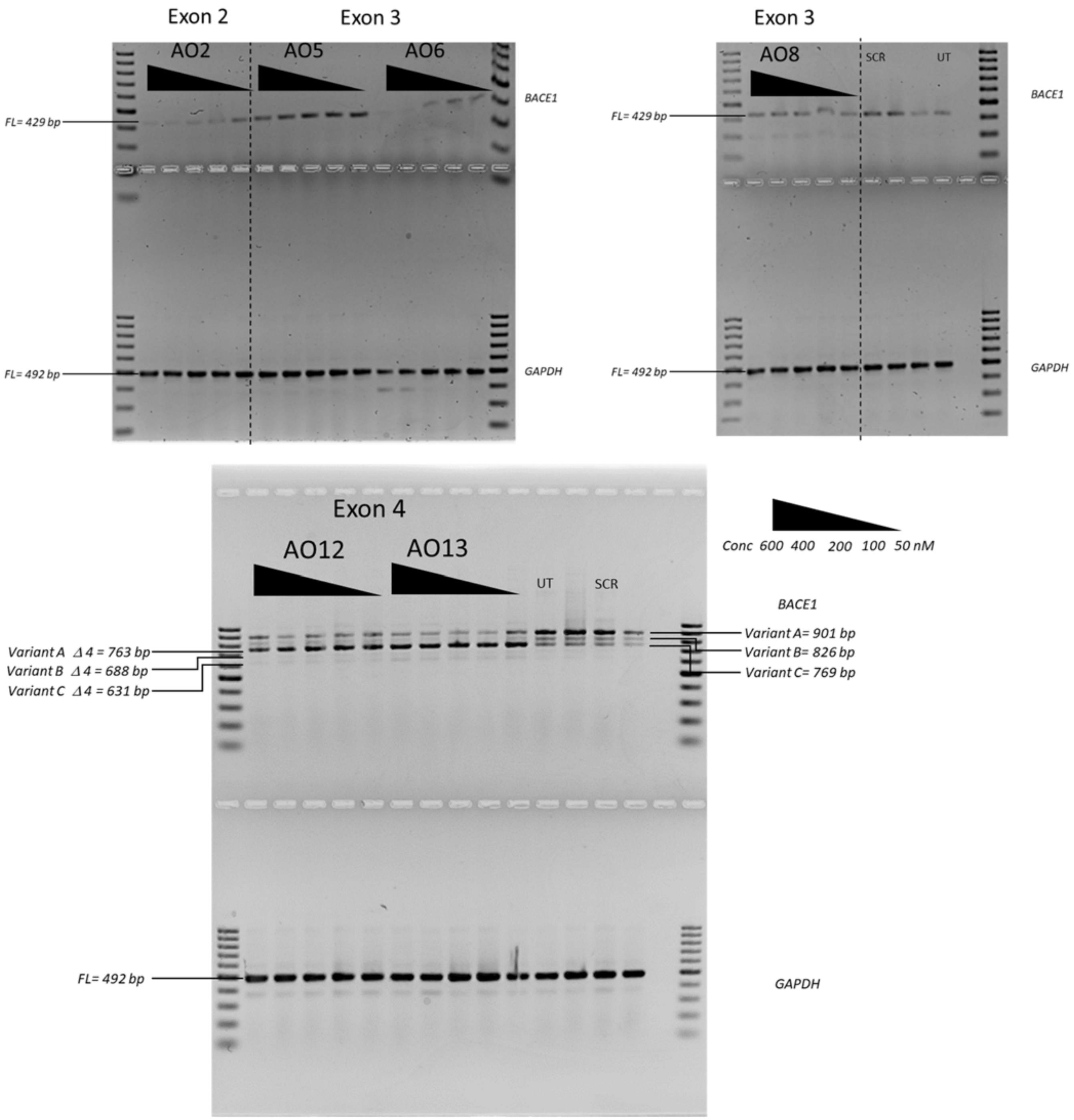

Figure S3. The RT-PCR products after treatment with AO2, AO5, AO6, AO8, AO12 and AO13. FL, full-length; *GAPDH* was used as a loading control; SCR, Scrambled or Gene tools control was used as a control. [The gel in this figure is the original gel representing the gel in Figure 2 of the article. The cropped gel has been shown in Figure 2 of the article due to other unimportant samples that exist between the desired samples.].

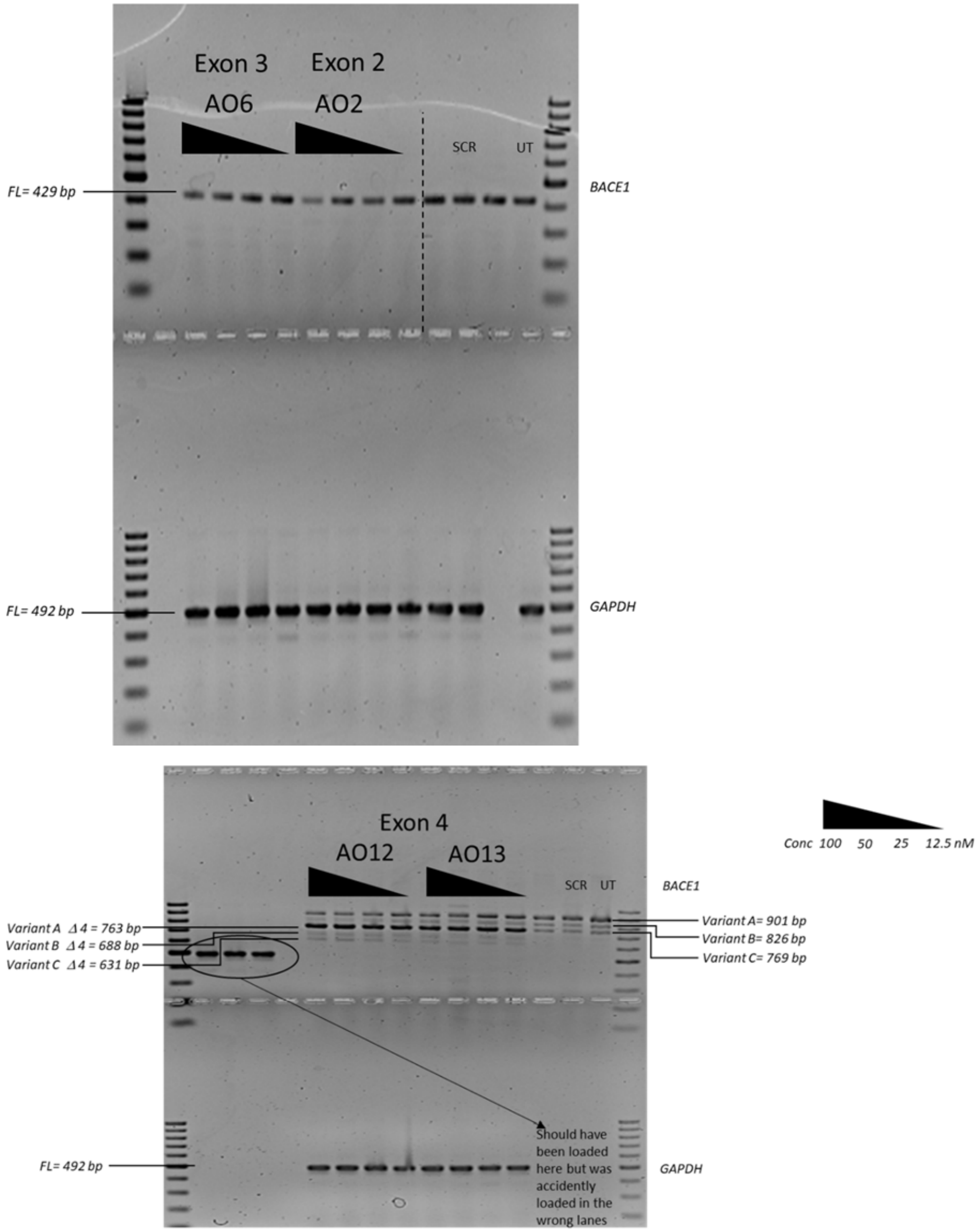

Figure S4. The RT-PCR products after treatment with AO2, AO6, AO12, and AO13. FL, full-length; *GAPDH* was used as a loading control; SCR, Scrambled or Gene tools control was used as a control. [The gel in this figure is the original gel representing the gel in Figure 3 of the article. The cropped gel has been shown in Figure 3 of the article due to other unimportant samples that exist between the desired samples and the samples loaded in the wrong wells.].

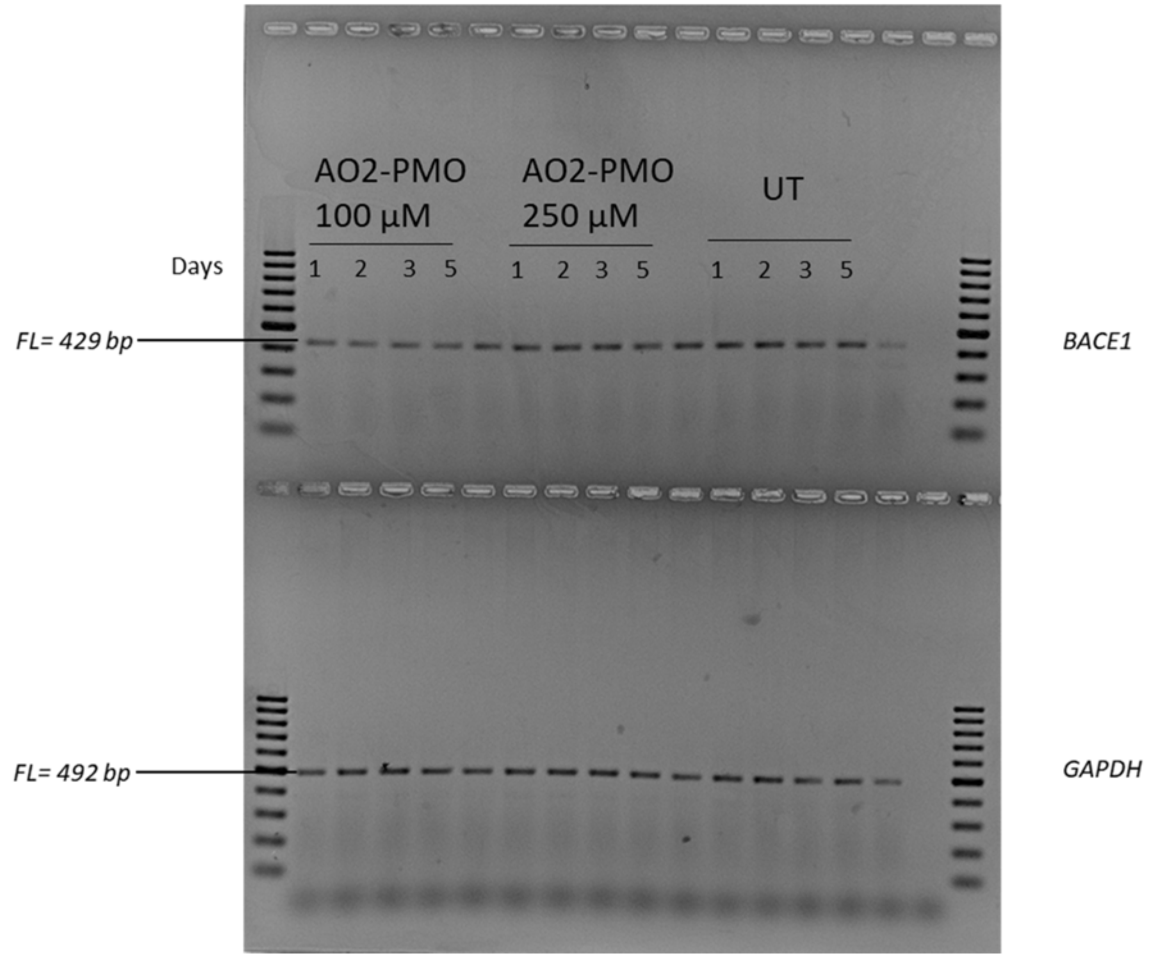

Figure S5. The RT-PCR products after treatment with AO2-PMO. FL, full-length; *GAPDH* was used as a loading control SCR, Scrambled or Gene tools control was used as a control. [The gel in this figure is the original gel representing the gel in Figure 5 of the article. The cropped gel has been shown in Figure 5 of the article due to other unimportant samples that exist between the desired samples.].

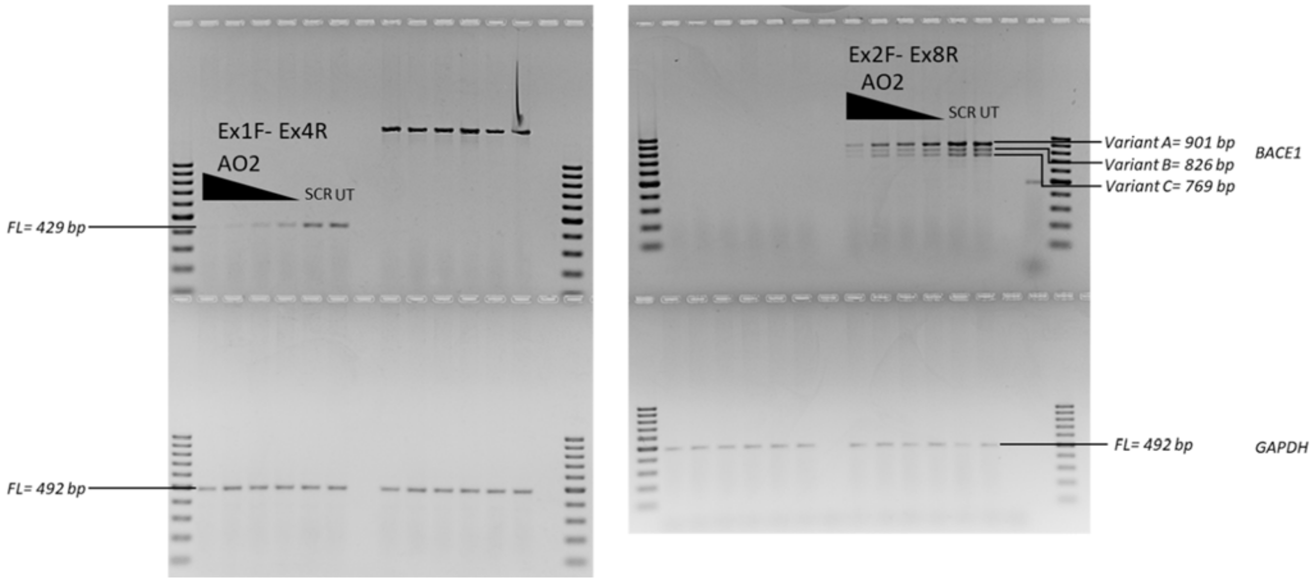

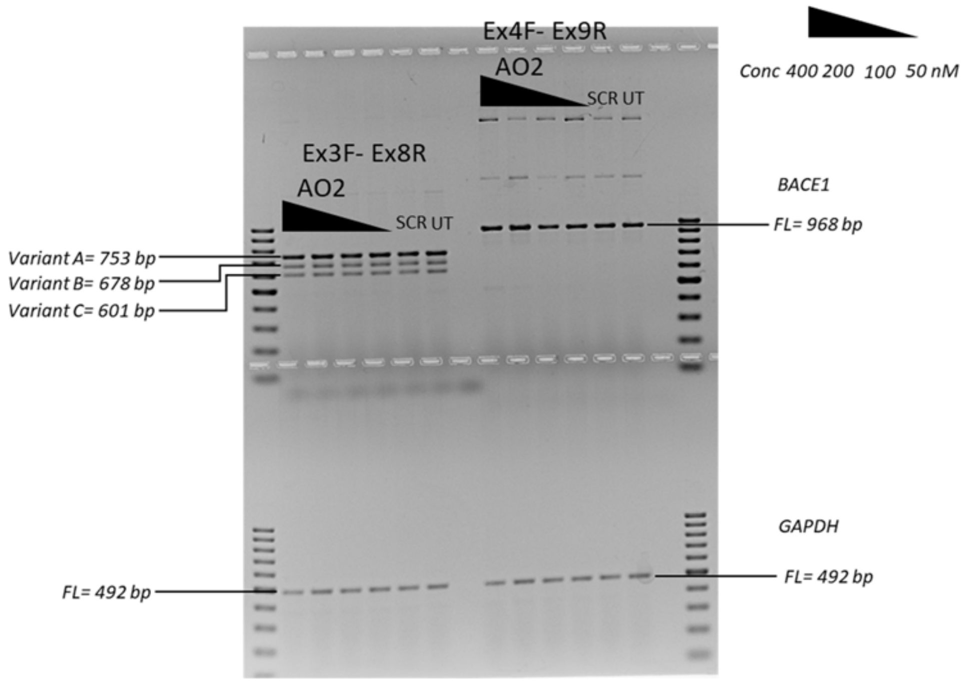

Figure S6. The RT-PCR products after AO2 treatment amplified using different primer sets. FL, full-length; *GAPDH* was used as a loading control; SCR, Scrambled or Gene tools control was used as a control. [The gel in this figure is the original gel representing the gel in Figure 6 of the article. The cropped gel has been shown in Figure 6 of the article due to other unimportant samples that exist between the desired samples and nonspecific bands that exist.].

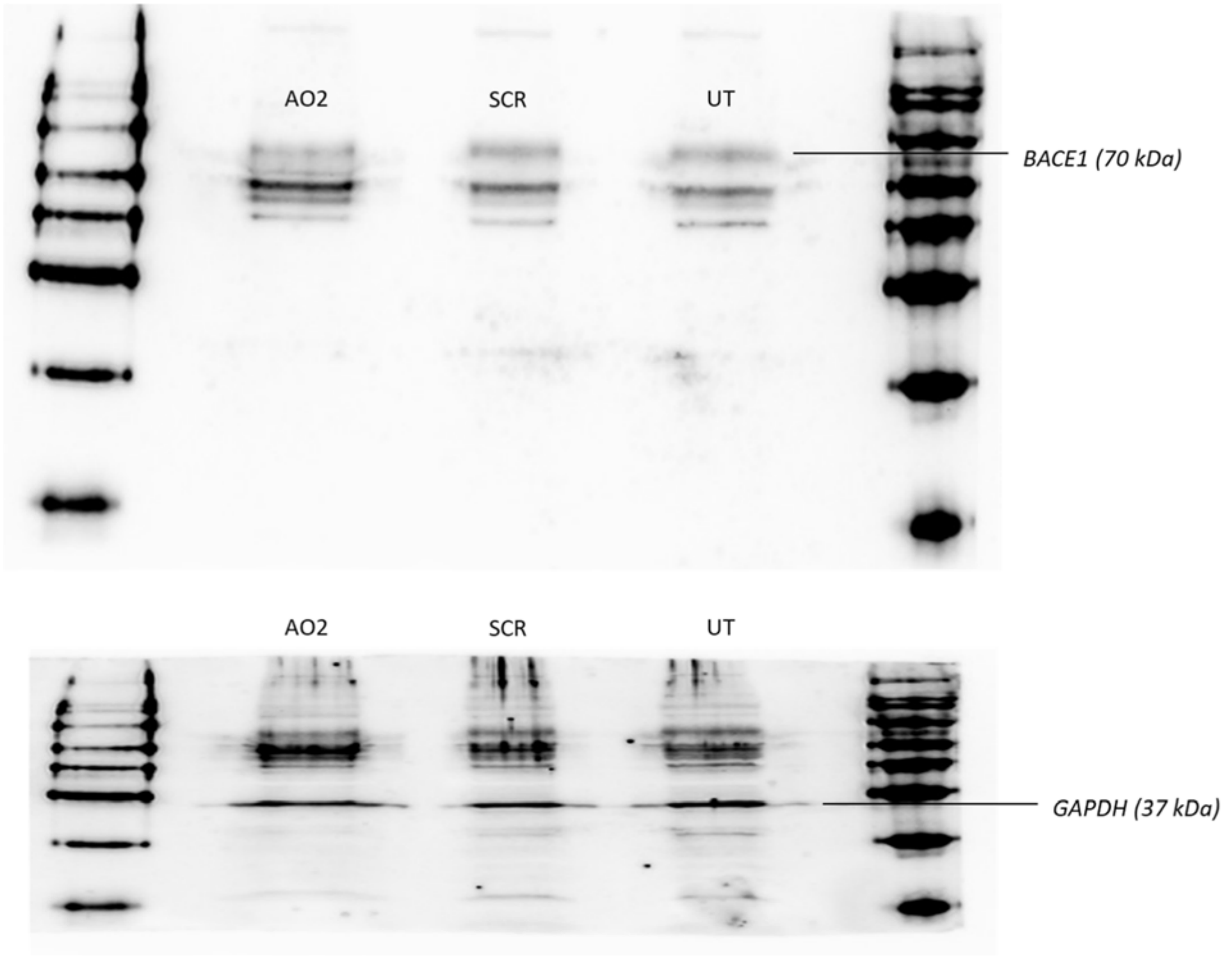

Figure S7. The western blot membranes after AO2 treatment incubated with anti-BACE1 antibody (top membrane) and anti-GAPDH antibody (bottom membrane). *GAPDH* was used as a loading control; SCR, Scrambled or Gene tools control was used as a control. [The membrane in this figure is the original membrane representing the membrane in Figure 7 of the article. The cropped membrane has been shown in Figure 6 of the article due to other nonspecific bands that exist.].

Methods:

Table S1. The seeding density of HEK293 cells used for different assays.

Assay	Plate or Flask?	Seeding density
RNA Extraction	24 well plate	50,000 cells/well
Western Blot	T25cm^2 flask	625,000 cells/flask
Nucleofection	24 well plate	100,000 cells/well

Table S2. The primer sets used to amplify *BACE1* transcript.

Primer sets	Primer pairs	Primer Sequences	Expected size
**Primer Set 1**	BACE1_Ex1Fa<br>BACE1_Ex4R	5′ GACAACCTGAGGGGCAAGTC 3′<br>5′ AACGTGGGTCTGCTTTACCA 3′	429 bp
**Primer Set 2**	BACE1_Ex2F<br>BACE1_Ex8R	5′ ACCAAAGTGAACCACGGAGG 3′<br>5′ TCTGGTAAAGCAGACCCACG 3′	968 bp
**Primer Set 3**	BACE1_4F<br>BACE1_Ex9R	5′ GGCAGCAGTAACTTTGCAGT 3′<br>5′ CCATAACAGTGCCCGTGGAT 3′	Variant A= 901 bp<br>Variant B= 826 bp<br>Variant C= 769 bp
**Primer Set 4**	BACE1_Ex3F<br>BACE1_Ex8R	5′ ACCTGGTAAGCATCCCCCAT 3′<br>5′ TCTGGTAAAGCAGACCCACG 3′	Variant A= 753 bp<br>Variant B= 678 bp<br>Variant C= 601 bp

Table 3. The primer sets used to amplify *GAPDH* transcript.

GAPDH Primer set	Primer pairs	Primer Sequences	Expected product length
**Primer Set 1**	GAPDH For<br>GAPDH Rev	5′ GGACTCATGACCACAGTCCATGC 3′<br>5′ TTACTCCTTGGAGGCCATGTGGG 3′	492 bp

Table 4. The PCR conditions for each primer set.

Primer pairs	PCR Conditions		
**BACE1 Primer set (25 ng each) BACE1_Ex1Fa BACE1_Ex4R**	**Temperature**	**Time**	
	55°C	30 min	
	94°C	2 min	
	94°C	30 s	30 cycles
	58°C	1 min	
	68°C	2 min	
**BACE1 Primer set (25 ng each) BACE1_Ex2F BACE1_Ex8R**	Temperature	Time	
	55°C	30 min	
	94°C	2 min	
	94°C	30 s	30 cycles
	55°C	1 min	
	68°C	2 min	
**BACE1 Primer set (25 ng each) BACE1_4F**	Temperature	Time	
	55°C	30 min	
	94°C	2 min	

**BACE1_Ex9R**	94°C	30 s	28 cycles
	60°C	1 min	
	68°C	2 min	
**BACE1 Primer set (25 ng each) BACE1_3F BACE1_Ex8R**	**Temperature**	**Time**	
	55°C	30 min	
	94°C	2 min	
	94°C	30 s	30 cycles
	60°C	1 min	
	68°C	2 min	
**GAPDH Primer set (12.5 ng each) GAPDH For GAPDH Rev**	**Temperature**	**Time**	
	55°C	30 min	
	94°C	2 min	
	94°C	30 s	18 cycles
	60°C	1 min	
	68°C	2 min	

Table 5. The HPLC analysis of the most efficient AOs (AO2, AO5, AO6, AO8, AO12, and AO13). All the AO samples were run on a Ion-Exchange HPLC using (1M NaClO4, 25mM Tris-HCl pH 8 and water) as mobile phase.

**Sample code**	**IE-HPLC chromatogram**
AO2	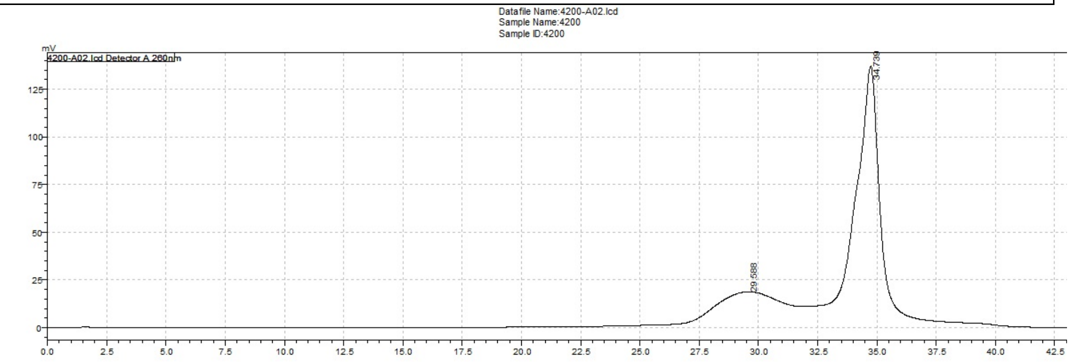
AO5	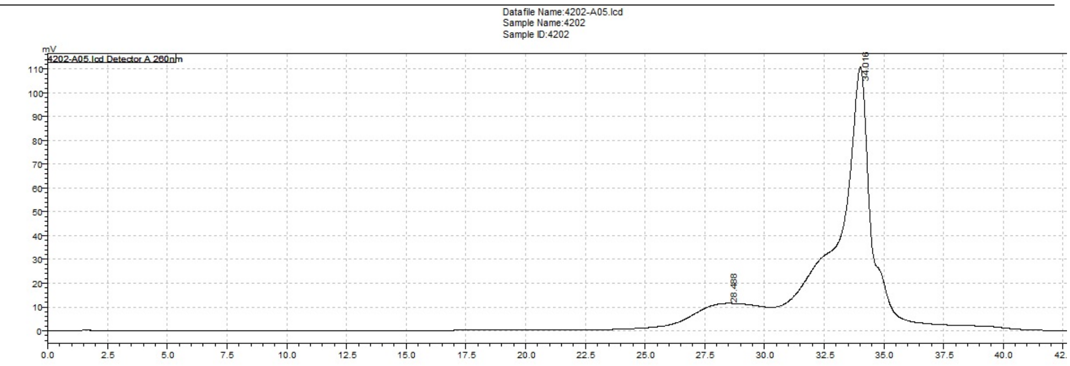
AO6	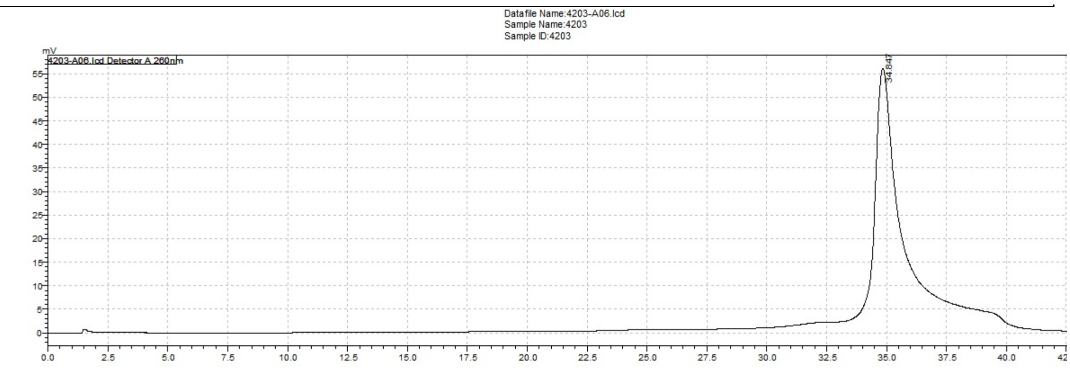

AO8	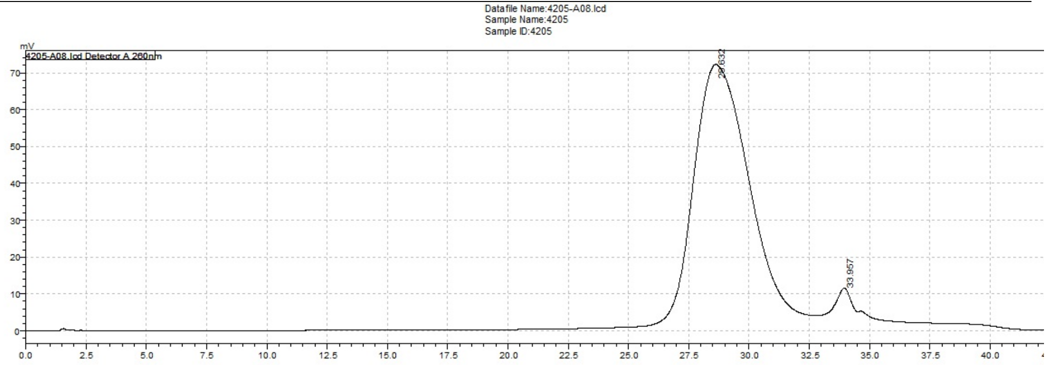
AO12	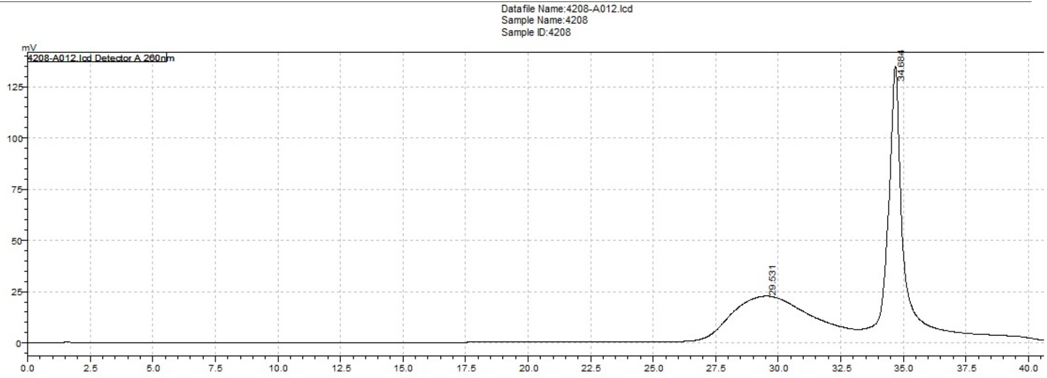
AO13	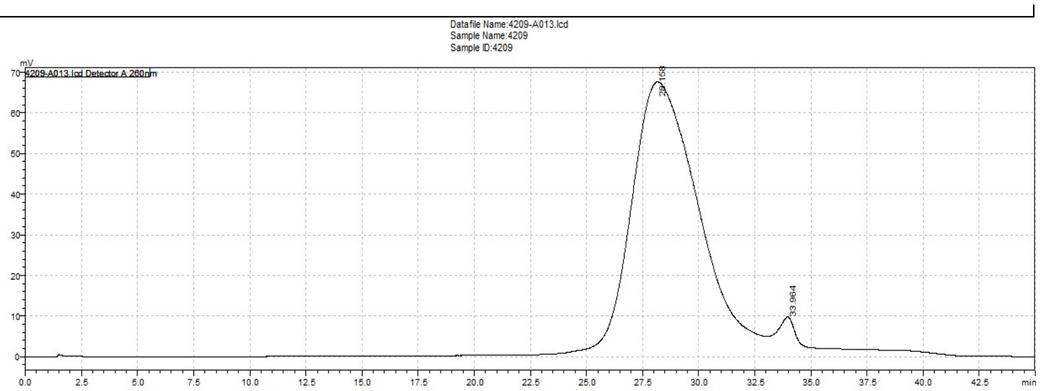
